# What Is the Role of Psychological Factors in Long COVID Syndrome? Latent Class Analysis in a Sample of Patients Recovered from COVID-19

**DOI:** 10.3390/ijerph20010494

**Published:** 2022-12-28

**Authors:** Giuseppe Craparo, Valentina Lucia La Rosa, Elena Commodari, Graziella Marino, Michela Vezzoli, Palmira Faraci, Carmelo Mario Vicario, Gabriella Serena Cinà, Morena Colombi, Giuseppe Arcoleo, Maria Severino, Giulia Costanzo, Alessio Gori, Ernesto Mangiapane

**Affiliations:** 1 Faculty of Human and Social Sciences, Kore University of Enna, 94100 Enna, Italy; 2 Department of Educational Sciences, University of Catania, 95124 Catania, Italy; 3 IRCCS—Referral Cancer Center of Basilicata (CROB), 85028 Rionero in Vulture, Italy; 4 Department of Psychology, University of Milano-Bicocca, 20126 Milan, Italy; 5 Department of Cognitive Sciences, Psychology, Education and Cultural Studies, University of Messina, 98122 Messina, Italy; 6 Department of Psychology, U.O.C., Azienda Sanitaria Provinciale Trapani, 91100 Trapani, Italy; 7 #LongCovid Facebook Group, 00118 Rome, Italy; 8 Pneumology Unit, Cervello Hospital, 90146 Palermo, Italy; 9 Associazione Orizzonti Onlus, 90121 Palermo, Italy; 10 Department of Health Sciences, University of Florence, 50121 Florence, Italy

**Keywords:** COVID-19, long COVID-19, emotional dysregulation, trauma

## Abstract

*Background*: This study aimed to identify clusters of long COVID-19 symptoms using latent class analysis and investigate the psychological factors involved in the onset of this syndrome. *Method:* Five hundred and six subjects recovering from COVID-19 completed a series of standardized questionnaires to evaluate the personality traits, alexithymia, and post-traumatic stress. *Results*: Five classes were identified: *Brain fog* (31.82%), *No symptoms* (20.95%), *Sensory disorders* (18.77%), *Breath impairment* (17.59%), and *Multiple disorders* (10.87%). Women reported post-COVID-19 respiratory symptoms and multiple disorders to a greater extent than men. Hospitalized subjects were more likely to report persistent symptoms after COVID-19 than asymptomatic or home-treated subjects. Antagonism, hyperarousal, and difficulty identifying emotions significantly predicted post COVID-19 symptoms. *Conclusions*: These findings open new questions for research on long COVID-19 and how states of emotional dysregulation can alter the physiological processes of the body and contribute to the onset of organic pathologies.

## 1. Introduction

Starting with the first case recorded in Wuhan, China in December 2019, the COVID-19 disease spread rapidly worldwide [1]. Globally, various countries have implemented severe restriction measures to contain the spread of the disease since the World Health Organization declared it a pandemic on 11 March 2020 [2]. According to the most recent estimates, 491 million people worldwide have been infected, more than six million have died from the consequences of the disease, and approximately 426 million have recovered [3].

Nearly 15 million people have been infected in Italy, and 160,000 have died from COVID-19 [3,4]. On 4 April 2022, Italy’s number of active infections was 1,284,016, while more than 13 million people have recovered since the onset of the pandemic [4,5].

Various patterns of disease severity can result from COVID-19 infections including asymptomatic infection and mild, moderate, severe, and critical infection [6,7]. Mild disease is characterized by minor symptoms (e.g., fever, cough, congestion or runny nose) without imaging features of pneumonia. Moderate infection presents with respiratory symptoms and imaging features of pneumonia. Finally, severe and critical infections are characterized by severe respiratory failure that requires the patient to be admitted to the intensive care unit (ICU) [6,8]. Approximately 10% to 15% of COVID-19 patients develop severe conditions, while the majority have mild to moderate symptoms. About 5% of patients have a critical clinical condition, resulting in ICU admission and often death [9,10].

Acute infection symptoms include fever, cough, dyspnea, tachypnea, and fatigue, often requiring supplemental oxygen to restore oxygen saturation [11,12,13]. In addition, COVID-19 can also cause symptoms in other organs, in addition to the respiratory system such as cardiac injury, thrombosis, kidney failure, gastrointestinal problems, and neurological impairments [13,14].

Despite the widespread and well-documented clinical manifestations of COVID-19 [15], long-term consequences remain unclear [16,17]. In fact, although the average recovery time from the disease is estimated to be approximately 2–3 weeks [10,18], one in five people may show symptoms for more than 5 weeks, while one in ten may show symptoms for more than 12 weeks [10,19]. For example, 55% of people who recovered from COVID-19 showed at least three persistent symptoms in Italy, and 32% of people had one or two persistent symptoms [20].

Following the recovery from COVID-19, chronic fatigue, diffuse myalgia, dyspnea, headache, loss of taste and smell, and difficulty with concentration are the most commonly reported symptoms [10,17,20], with a consequent significant impairment of physical, cognitive, and psychosocial health [17,21].

There is no consensus on the appropriate terminology to indicate this clinical condition that persists beyond the acute phase of infection [22]. In this regard, the term ‘long COVID-19’ has recently been proposed [23,24] and is gradually being adopted in the scientific literature [25,26]. Specifically, according to the National Institute of Health and Care Excellence guidelines, long COVID-19 is a clinical condition that includes both ongoing symptomatic COVID-19 infection, where symptoms last 4 to 12 weeks, and post-COVID-19 symptoms that can persist beyond 12 weeks after recovery [10,26,27].

There is extensive literature on the psychological consequences of COVID-19 in patients recovering from the disease [28,29,30,31]. In particular, there is a significantly higher likelihood of developing post-traumatic stress disorder (PTSD) among patients with more severe symptoms associated with COVID-19. Specifically, intrusion and hyperarousal symptoms are more common in patients admitted to the ICU for acute COVID-19 treatment [32,33]. In a recent study, Craparo et al. [34] found that people who recovered from COVID-19 had high levels of alexithymia, dissociation, anxiety, and depression, which significantly predicted the three main clusters of PTSD symptoms (i.e., avoidance, intrusion, and hyperarousal). Furthermore, post-traumatic symptoms were more likely to develop following COVID-19 in individuals with dysfunctional personality traits such as negative affectivity and psychoticism [34].

Despite growing interest in the psychological consequences of COVID-19 in individuals who recovered from the disease, there is limited research investigating the relationship between the persistent symptoms of long COVID-19 and individual personality characteristics. Given that long COVID-19 is a clinical condition that will affect millions of people worldwide [22], it is essential to investigate the psychological factors associated with this condition to identify possible risk factors and the most effective intervention strategies to improve the quality of life and psychological well-being of these patients.

In light of these considerations, this study aimed to identify clusters of long COVID-19 symptoms and understand whether different classes are associated with individual-level constructs (i.e., personality traits, alexithymia, and post-traumatic disorder). In particular, a significant association with long COVID-19 symptoms was hypothesized to exist between alexithymia, post-traumatic stress, and dysfunctional personality traits (e.g., negative affectivity, detachment, antagonism, disinhibition, and psychoticism).

## 2. Materials and Methods

This study was conducted between May and July 2021 as part of a large longitudinal cross-sectional project.

Participants were Italian individuals with an official diagnosis of COVID-19 who recovered from the disease and had undergone different options for the treatment.

An online survey was designed using Google Forms and disseminated through the main social media platforms (Facebook, Instagram, and Twitter). In addition, the survey was sent to patients attending COVID-19 centers participating in this study. The completion of the online questionnaire was set up to ensure the confidentiality of the data collected and no sensitive data that could identify the participants were recorded.

The inclusion criteria for the study were: being older than 18 years old, having received an official diagnosis of COVID-19 by swab positivity, being recovered from COVID-19 and testing negative for a swab, and having expressed consent to participate in the research. People who answered that they had not contracted COVID-19 at the time of filling out the questionnaire were automatically stopped from continuing to answer the questions. Furthermore, other exclusion criteria were the presence of psychiatric comorbidities and the assumption of psychopharmacological medications assessed through specific screening questions.

The survey was completed anonymously, voluntarily, and without any remuneration. Participants were informed of the purpose of the study and provided their online consent to participate. Furthermore, they were informed that they could withdraw without consequence at any time during the study.

All procedures were carried out following the Declaration of Helsinki and the Internal Review Board for psychological research of the University of Enna Kore approved the study protocol (1 April 2021).

### 2.1. Measures

The online survey consisted of three sections. In the first section, socio-demographic information was collected concerning sex, age, education level, and employment status. In the second section of the survey, participants indicated whether they had contracted COVID-19, what type of treatment they were given (none, home therapy, ordinary hospitalization, admission to the ICU), and whether there were any family members who were affected or bereaved by the disease. Furthermore, they were asked to report persistent symptoms after recovery from COVID-19. The symptoms reported by at least 5% of the participants were included in the analysis. The third section included a battery of standardized questionnaires designed to measure a variety of psychological constructs. For this study, we considered the personality traits, post-traumatic stress symptoms related to COVID-19, and alexithymia. In the following paragraphs, we describe the scales used in this study. A full description of the questionnaires used in the research can be found in our previous article [34].

#### 2.1.1. Personality Inventory for DSM-5 Brief Form (PID-5-BF)

Starting from the classification of personality disorders reported in the fifth edition of the Diagnostic and Statistical Manual of Mental Disorders (DSM-5) [35], the DSM-5 Personality and Personality Disorders Workgroup [36] promoted the development of a model of maladaptive personality traits. Specifically, the model included five personality domains: negative affectivity, detachment, antagonism, disinhibition, and psychoticism [35,37]. According to the DSM-5 classification [35], anxiety, separation insecurity, and emotional lability are characteristics of negative affectivity; detachment is defined as withdrawal, anhedonia, and avoidance of intimacy; antagonistic traits include manipulation, deception, and grandiosity; disinhibition includes irresponsibility, impulsivity, and distraction; and psychoticism is characterized by eccentricity and unusual beliefs and experiences.

The PID-5-BF [38] is a 25-items self-reporting questionnaire that assesses the personality traits described above. Respondents are asked to answer each item (e.g., “People would describe me as reckless”, “I feel like I act totally on impulse”) on a four-point Likert scale ranging from 0 (*very false or often false*) to 3 (*very true or often true*). The Italian adaptation of the scale was used in this study [39]. In our sample, the scale showed a good level of reliability (negative affectivity: α = 0.75, detachment: α = 0.75, antagonism: α = 0.72, disinhibition: α = 0.74, psychoticism: α = 0.78).

#### 2.1.2. Impact of Event Scale-Revised (IES-R)

The IES-R [40] is a self-reporting questionnaire containing 22 items designed to assess post-traumatic symptomatology following an unexpected exposure to death, a threat to life, or a threat to physical or mental integrity [41,42].

The questionnaire allows one to calculate the a total post-traumatic stress score and the scores of the three subscales that correspond to the clusters of PTSD (i.e., avoidance, intrusion, and hyperarousal). Each item was rated on a five-point scale from 0 (*not at all*) to 4 (*extremely*). The Italian version of the IES-R [43] was adapted for this study, referring to the specific COVID-19 disease event (e.g., “Any reminders brought back feelings about the COVID-19 disease”). The IES-R showed good reliability in our sample (avoidance α = 0.86, intrusion α = 0.91, and hyperarousal α = 0.74).

#### 2.1.3. Toronto Alexithymia Scale-20 (TAS-20)

TAS-20 is a self-reporting questionnaire consisting of 20 items used to evaluate alexithymia [44]. As a result of alexithymia, individuals are unable to recognize or describe emotions due to a lack of awareness of their emotional states. In addition, a person suffering from this condition also exhibits externally oriented thinking and poor imaginal abilities [45]. Each item (e.g., “I am often confused about what emotion I am feeling”, “It is difficult for me to find the right words for my feelings”) was rated on a five-point Likert scale from 1 (*strongly disagree*) to 5 (*strongly agree*). In addition to the total alexithymia score, it is possible to calculate scores for the three main dimensions of alexithymia: difficulty in identifying feelings (DIF), difficulty in describing feelings (DDF), and externally oriented thinking (EOT). The Italian version of the TAS-20 was used [46,47] with a good reliability (α = 0.95).

### 2.2. Statistical Analyses

A confirmatory factor analysis (CFA) was carried out to test the measurement model of each scale. A detailed description of the analyses was reported in our previous study [34].

Mean (M) ± standard deviation (SD) were used to express continuous variables, while frequencies and percentages were used to express the categorical variables. The scores for each scale were calculated as the average of the items.

Latent class analysis (LCA) was used to identify subgroups of subjects who recovered from COVID-19 with similar experiences with long COVID-19 symptoms (i.e., latent classes). Based on the similarities between the participants’ responses and those of other participants in the sample, LCA assigns them probabilistic likelihoods of membership in a predefined number of classes. This technique highlights the manifest response patterns that may suggest latent typologies underlying manifest responses.

For a more thorough description of LCA, refer to McCutcheon [48] and Collins and Lanza [49]. The input for the analysis was the following list of symptoms: cough; chest pain; fatigue; respiratory difficulties; anosmia; dysgeusia; hair loss; cognitive, sleep, cardiovascular, hearing, skin, sight, intestinal, hormonal, neurological, psychological, vasomotor disorders; myalgia; headache.

Each class model was fitted and compared separately using the Akaike information criterion (AIC), the consistent AIC (CAIC), the Bayesian information criterion (BIC), and the adjusted BIC (ABIC). In this regard, Nylund et al. [50] conducted an extensive simulation study comparing many of these tools and found that among the information criteria, the aBIC was superior to alternatives such as the AIC, the CAIC, and the standard BIC. As a result, aBIC values were used to determine the final number of latent classes.

Differences among the latent classes in demographic, clinical, and psychological characteristics were evaluated using Chi-square and ANOVA tests. Finally, a multinomial logistic regression was run to identify the psychological variables that may constitute protective or risk factors for the manifestation of a class of long COVID-19 symptoms. All variables were standardized to z-scores to compare the regression coefficients. A *p*-value of < 0.05 was considered statistically significant.

All of the analyses were performed with R for macOS, version 4.0.5. [51]. LCA was conducted in the *poLCA* package [52], and the multinomial logistic regression was conducted using the nnet package [53].

## 3. Results

### 3.1. Sample

Five hundred and thirty respondents completed the online survey. Those who were affected by COVID-19 when taking the survey (*n* = 22) and did not complete the questionnaires (*n* = 2) were excluded. The final study sample included 506 participants. Table 1 shows the main characteristics of the sample.

### 3.2. Latent Class Analysis

Table 2 reports the fit indices for the potential classes identified. We opted for a 5-class model because it is characterized by the best adjustment and interpretability values compared to the other models. In particular, the aBIC value for the 5-class model was lower than the value for the other solutions.

**Figure 1:**
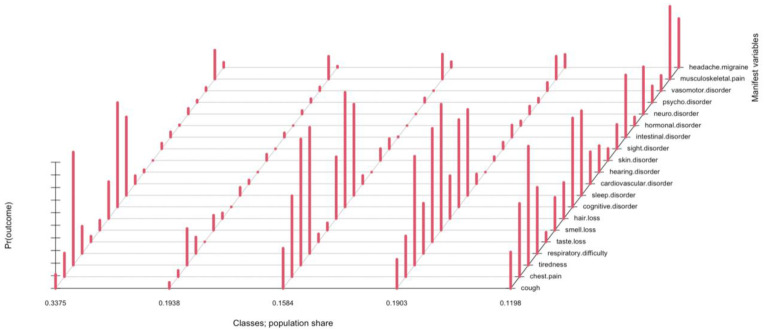
Composition of long COVID-19 symptoms in the latent classes.

Class names were chosen based on the long COVID-19 symptoms prevalent in each class. The first class (*Brain fog*) was characterized by cognitive impairment, fatigue, sleep disturbance; the second class (*No symptoms*) was composed of individuals who did not report significant post-COVID-19 symptoms; the third class (*Breath impairment*) was mainly characterized by chest pain and breathing disorders, associated with fatigue and sleep disturbances; the fourth class (*Sensory disorders*) was characterized in particular by anosmia and dysgeusia, together with fatigue, cognitive and sleep disorders; and the fifth class (*Multiple disorders*) was characterized by the presence of symptoms of different nature including intestinal disorders, headaches, myalgia, fatigue, and sleep disorders. Figure 1 shows in detail the five classes identified in relation to the symptoms of long COVID-19.

The majority of individuals was classified in the *Brain fog* class (*n* = 161; 31.82%), followed by *No symptoms* (*n* = 106; 20.95%), *Sensory disorders* (*n* = 95; 18.77%), *Breath impairment* (*n* = 89; 17.59%), and *Multiple disorders* (*n* = 55; 10.87%) classes.

### 3.3. Demographic, Clinical and Psychological Characteristics

We analyzed the difference between the five latent classes in terms of the sociodemographic, clinical (Table 3), and psychological variables (Table 4) investigated in the study.

A chi-square test of independence was performed to examine the relationship between gender and symptom classes. The relation was significant (χ^2^ (4) = 15.44, *p* = 0.003, V = 0.17) and the computed effect size suggests a strong effect. In particular, women are more likely to experience long COVID-19 breath impairment and multiple disorders symptoms than men.

We also found a medium-to-large significant association between the type of therapy for COVID-19 and the symptom classes (χ^2^ (16) = 37.76, *p* = 0.002, V = 0.14). Specifically, asymptomatic people and those undergoing home treatment were more likely to have no post COVID-19 symptoms or primarily cognitive symptoms. Conversely, people hospitalized in ordinary regimen or sub-intensive care tended to experience more post-COVID-19 cognitive or respiratory symptoms. Finally, individuals who had been admitted to the ICU were more likely to experience cognitive, respiratory, and sensory symptoms. ANOVA one-way tests were performed to determine whether the mean age and mean days of hospitalization were different among the classes of symptoms. No significant differences were found in relation to age (F(4, 501) = 0.94, *p* = 0.439, η^2^ = 0.007). In contrast, we found a small-to-medium difference for days of hospitalization (F(4, 501) = 2.97, *p* = 0.019, η^2^ = 0.023).

Table 4 reports the ANOVA one-way test results in terms of the differences between the five classes related to personality traits, alexithymia, and post-traumatic stress levels.

### 3.4. Multinomial Logistic Regression

Multinomial logistic regression was performed to assess the impact of a number of psychological variables on the likelihood that individuals who recovered from COVID-19 would report that they experienced a specific class of long COVID-19 symptoms. PID-5-BF, TAS-20, and IES-R sub-factors were considered independent variables. The reference category, chosen for the multinomial logistic regression model, was the *No symptoms* class.

As shown in Table 5, hyperarousal was a strong predictor of *Brain fog* (OR = 2.54, *p* < 0.001), *Breath impairment* (OR = 2.33, *p* = 0.01), and *Sensory disorders* (OR = 2.16, *p* = 0.01) classes.

Furthermore, difficulty in identifying emotions was a risk factor for the *Multiple disorders* (OR = 3.87, *p* < 0.001), *Breath impairment* (OR = 3.17, *p* < 0.001), and *Brain fog* (OR = 2.05, *p* < 0.001) classes.

Regarding personality traits, antagonism was found to be a significant risk factor for *Brain fog* (OR = 0.64, *p* = 0.01), *Breath impairment* (OR = 0.65, *p* = 0.04), and *Sensory disorders* (OR = 0.66, *p* = 0.03) classes.

## 4. Discussion

This study aimed to identify classes of individuals based on persistent symptoms reported after recovery from COVID-19 and to evaluate any risk factors for developing these symptoms with particular attention to the personality characteristics.

The results of this study highlighted many interesting elements that both confirm the literature already available on this topic and open new questions for future research.

First, in accordance with the literature [10,14], we found statistically significant differences between men and women in the development of long COVID-19 syndrome symptoms. More specifically, women tended to present with post-COVID-19 respiratory and multiple disorders symptoms to a greater extent than men. This finding confirms the results of a Spanish multicenter study, according to which women are at a higher risk of developing long-term post-COVID-19 symptoms including dyspnea, fatigue, pain, ocular problems, and sleep disorders [54]. Furthermore, according to Righi et al. [17], women are at higher risk of psychological distress after COVID-19, confirming the different psychological impact of the disease on the quality of life of women. This result is significant because it underlines the need for a gender medicine that considers the differences between men and women concerning long COVID-19 symptoms to promote individualized care [54].

Our study also showed that asymptomatic people and those undergoing home treatment were more likely to report no post COVID-19 symptoms or at most cognitive symptoms. Conversely, people hospitalized in ordinary regimen or sub-intensive care tended to experience more post-COVID-19 cognitive or respiratory symptoms. Finally, subjects admitted to the ICU were more likely to experience cognitive, respiratory, and sensory symptoms. Furthermore, we found a significant difference between the classes related to the length of hospitalization. These findings are consistent with previous results showing the significant impact of the type of therapy for COVID-19 treatment and the length of hospitalization on the presence of persistent symptoms after recovery [22]. Indeed, as shown in the study by Righi et al. [17], the most severe forms of illness requiring hospitalization and, in extreme cases, admission to the ICU, were associated with an increased risk of symptom persistence for up to 9 months after COVID-19 recovery. Similarly, Arnold et al. [55] reported that 59% of COVID-19 patients with a mild disease continued to have COVID-19 symptoms 8–12 weeks after symptom onset. In addition, 75% of patients with moderate acute symptoms and 89% of patients with severe symptoms requiring oxygen supplementation and/or admission to the ICU had persistent symptoms within the same period [55]. Finally, according to the literature, a lengthy hospitalization is significantly associated with the development of persistent symptoms after recovery from COVID-19 [10,17,55].

Therefore, based on these findings, it is essential to ensure that patients cured of COVID-19, particularly those hospitalized, have regular follow-ups to assess the persistence of symptoms after recovery to improve their clinical outcomes [10].

In contrast, unlike our expectations, there were no significant differences between symptom classes concerning age in our study sample. Indeed, many studies underlined that older age is a risk factor for developing persistent symptoms related to COVID-19 [10,17,55]. A possible reason for this discrepancy might be that the average age of our sample was relatively low. Consequently, it is not possible to assess the true impact of this variable on the presence of persistent symptoms after COVID-19.

We have highlighted some findings worthy of attention about the psychological variables that were the subject of our study.

Alexithymia, particularly difficulty in identifying emotions, has been found to be a significant risk factor for the onset of post-COVID-19 respiratory, cognitive, and multi-organ symptoms. In the literature related to this psychological construct, there is much debate about how alexithymia may be involved in the development and prognosis of organic diseases. Several hypotheses have been formulated regarding four mediating pathways: physiological, behavioral, cognitive, and social [56]. A first possible explanation is that the impaired capacity for emotional regulation typical of alexithymic individuals might produce alterations in the autonomic, endocrine, and immune systems that, in turn, result in the destruction of the body’s homeostasis with the consequent onset of organic diseases [57,58]. Second, alexithymia is a risk factor for adopting unhealthy behaviors (i.e., alcohol and drug use, a sedentary lifestyle) because of the difficulty of alexithymic subjects to cope adaptively to stressful life events [45,58,59,60]. Third, alexithymic subjects have problems recognizing their physical and emotional states, thus making diagnosis and prognosis of their organic disorders particularly difficult [56,58]. Finally, from a social point of view, alexithymic individuals cannot recognize and understand their own and others’ emotional states. This difficulty does not allow them to take advantage of a good social support network to cope with stress [57,58]. In addition, it is not yet clear whether alexithymia can be considered as a cause or an effect of the onset of organic diseases. In this regard, the significant effects of this variable on the clinical outcomes of patients suffering from a wide variety of conditions including respiratory disorders [61], cardiac diseases [62], gastrointestinal disorders [63], and cancer [64] have been extensively documented.

Considering these data, a possible interpretation of our findings is that alexithymia contributes to the increased traumatic impact of the COVID-19 experience, as underlined in the study by Craparo et al. [34]. Consequently, according to the hypotheses of the literature, a state of emotional dysregulation may contribute to further compromise of the balance of the organism’s physiological processes under stress due to COVID-19 disease, and thus favor the onset of persistent symptoms after recovery. Obviously, given the novelty of this finding, further studies with larger samples will be necessary to verify and confirm the role of alexithymia in the onset and maintenance of long COVID-19 syndrome.

Another interesting finding of our study concerns the significant role of hyperarousal in the onset of a variety of post COVID-19 symptoms (cognitive, respiratory, and sensory). As other studies have already shown [28,32,33,34], high levels of post-traumatic stress and an increased risk of developing PTSD were reported in patients who recovered from COVID-19. In this regard, it is widely demonstrated that a central feature of PTSD refers to a discrepancy between physiological states and the psychological or behavioral processes required to adapt appropriately to environmental conditions. Specifically, patients with PTSD experience abnormal oscillations in autonomous states that support combat and flight behaviors, or withdrawal, immobilization, and dissociation. According to Williamson et al. [65], this impairment of the autonomic function in response to social and environmental demands is a core element of PTSD.

Moreover, as confirmed by studies based on Porges’ polyvagal theory [66,67], which emphasizes the fundamental role of the autonomic nervous system in explaining the brain–body connection, changes in autonomic function contribute to the onset of organic disorders of various kinds, in particular, brain dysfunction and cardiovascular disease [68]. Thus, the state of chronic stress and hyperarousal that characterizes PTSD is associated with poor health outcomes. For example, it has been shown that, after the World Trade Center attacks, survivors who had developed PTSD were at higher risk for diabetes [69] and cardiovascular diseases [70].

In light of these considerations, we can hypothesize that the state of hyperarousal that characterizes the post-traumatic stress caused by the experience of COVID-19 disease may alter the functioning of the autonomic nervous system and the balance of physiological processes of the body. Thus, this imbalance at the physiological level could promote the onset and persistence of physical symptoms even after recovery from COVID-19. Future studies with larger samples should further test this hypothesis, which may have important implications for treating long COVID-19 syndrome.

Finally, we found significant differences between the classes in relation to personality traits. More specifically, negative affectivity, detachment, and psychoticism scores were significantly higher in individuals with post-COVID-19 symptoms than those without these symptoms. Furthermore, antagonism seemed to be a significant risk factor for developing long COVID-19 syndrome. These results are in line with those of our previous study, which showed that negative affectivity and psychoticism appear to predict the development of PTSD after recovery from COVID-19 [34]. In particular, antagonism is configured as a personality trait that increases the risk of developing persistent symptoms after recovery from COVID-19. This finding is certainly very interesting, and it can be interpreted in light of the literature data. According to the DSM-5 [35], antagonism is characterized by “behaviors that put the individual at odds with other people including an exaggerated sense of self-importance and a concomitant expectation of special treatment” (p. 780). Recent studies investigating the relationship between personality traits and COVID-19 compliance showed that individuals with higher levels of antagonism reported less compliance with preventive measures against COVID-19 [71,72,73]. Furthermore, antisocial personality traits such as antagonism have also been shown to be associated with greater hesitancy toward COVID-19 vaccination [74]. In light of these data, we can hypothesize that individuals with antisocial personality traits are also more at risk of developing post-COVID-19 symptoms due to lower compliance with medical treatments as well as with vaccination, which reduces the risk of persistent symptoms after recovery from COVID-19.

### Strengths and Limitations

As far as we know, this is one of the first studies on the psychological factors involved in the onset of long COVID-19 syndrome, which now represents a significant public health problem. More specifically, the study used latent class analysis to identify clusters of subjects based on the symptoms they reported after recovery from COVID-19. These classes of subjects were then compared based on a series of socio-demographic, clinical, and psychological variables. Our results confirm the existing literature data on the topic and allowed us to formulate new hypotheses on the risk factors for this syndrome. Other important strengths are the considerable sample size of our study and the use of a validated battery of questionnaires that also demonstrated good psychometric characteristics in our sample.

This study also has some limitations to consider. The cross-sectional nature of the study did not allow us to test the exact causal relationship between variables; longitudinal studies with large samples are required for this purpose. Furthermore, online sampling did not allow us to balance the sample composition for variables such as gender, age, type of therapy, and length of hospitalization. Therefore, further studies with larger samples representative of the general population will be needed to assess the effect of these variables on the study outcomes.

## 5. Conclusions

This study highlights the need for attention to the long-term consequences of COVID-19 on both a physical and psychological level. In particular, the potential role of psychological factors such as alexithymia and post-traumatic stress in the onset and persistence of symptoms even after recovery from COVID-19 confirms the need for further research on the brain–body connection and how states of emotional dysregulation and stress can alter the body’s physiological processes and contribute to the onset of organic pathologies.

Furthermore, because trauma is stored in somatic memory and manifests in changes in the body’s response to stress, psychotherapy settings focusing on the experiences of the body are needed [34]. Thus, the COVID-19 pandemic may be an opportunity to implement new approaches to body-centered psychotherapeutic trauma treatment.

## Figures and Tables

**Table 1:** Characteristics of the sample.

Variable		Value
Gender	Female (%)	435 (86.0)
	Male (%)	71 (14.0)
Marital status	Single (%)	85 (16.8)
	Married (%)	289 (57.1)
	Live-in-partner (%)	57 (11.3)
	Separated (%)	31 (6.1)
	Divorced (%)	36 (7.1)
	Widowed (%)	8 (1.6)
Highest educational level	Primary school (%)	6 (1.2)
	Middle school (%)	70 (13.8)
	High school (%)	242 (47.8)
	Bachelor degree (%)	53 (10.5)
	Master degree (%)	89 (17.6)
	Post-graduate degree (%)	46 (9.1)
Employment	Unemployed (%)	44 (8.7)
	Seeking first employment (%)	3 (0.6)
	Student (%)	6 (1.2)
	Armed forces (%)	5 (1.0)
	Craftsman (%)	7 (1.4)
	Employee (%)	192 (37.9)
	Entrepreneur (%)	10 (2.0)
	Freelancer (%)	39 (7.7)
	Healthcare personnel (%)	86 (17.0)
	Housekeeper (%)	35 (6.9)
	Merchant (%)	10 (2.0)
	Religious (%)	1 (0.2)
	School personnel (%)	43 (8.5)
	Retired (%)	25 (4.9)
COVID-19 therapy	Asymptomatic (%)	34 (6.7)
	Domiciliary (%)	366 (72.3)
	Ordinary hospitalization (%)	62 (12.3)
	Sub-intensive care (%)	32 (6.3)
	Intensive care (%)	12 (2.4)
Family members affected by COVID-19	Yes (%)	342 (67.5)
	No (%)	164 (32.5)
Family deaths due to COVID-19	Yes (%)	54 (10.7)
	No (%)	452 (89.3)
Days of hospitalization (range: 1–118)	M ± SD	4.52 ± 12.20
Days of home isolation (range: 0–120)	M ± SD	32.22 ± 18.53

**Table 2:** Latent class profile solutions and fit indices.

Model	LL	BIC	ABIC	CAIC	LR
1 Class	−4054.05	8232.63	8169.14	8252.63	2742.23
2 Classes	−3856.62	7968.52	7838.39	8009.52	2347.37
3 Classes	−3750.52	7887.09	7690.29	7949.09	2135.18
4 Classes	−3690.95	7898.70	7635.25	7981.70	2016.03
5 Classes	−3657.95	7963.47	7633.37	8067.47	1950.05
6 Classes	−3635.54	8049.40	7652.64	8174.40	1905.22

Note. AIC = Akaike information criterion; BIC = Bayesian information criterion; ABIC = Adjusted Bayesian information criterion; CAIC = Consistent Akaike information criterion; LL = log-likelihood; LR = log-likelihood ratio.

**Table 3:** Symptom classes in terms of the sociodemographic and clinical characteristics (*N* = 506).

	No Symptoms *n* = 106	Brain Fog *n* = 161	Breath Impaired *n* = 89	Sensory Disorders *n* = 95	Multiple Disorders *n* = 55
**Gender**					
Male *n* (%)	24 (34%)	24 (34%)	5 (7%)	15 (21%)	3 (4%)
Female *n* (%)	82 (19%)	137 (32%)	84 (19%)	80 (18%)	52 (12%)
**Mean Age (SD)**	46.04 (12.70)	47.55 (10.79)	48.12 (9.91)	47.90 (10.33)	45.47 (10.19)
**Type of treatment**					
Asymptomatic *n* (%)	17 (50%)	11 (32%)	2 (6%)	2 (6%)	2 (6%)
Home treatment *n* (%)	78 (21%)	116 (32%)	56 (15%)	75 (21%)	41 (11%)
Ordinary hospitalization *n* (%)	7 (11%)	20 (32%)	18 (29%)	11 (18%)	6 (10%)
Sub-intensive care *n* (%)	3 (9%)	10 (31%)	10 (31%)	4 (12%)	5 (16%)
Intensive care *n* (%)	1 (8%)	4 (33%)	3 (25%)	3 (25%)	1 (8%)
**Mean Days of hospitalization (SD)**	2.86 (10.75)	3.86 (9.32)	8.12 (16.53)	3.37 (8.40)	5.84 (17.61)

Notes: The percentages of respondents were calculated by row, so each percentage represents the probability of belonging to the cluster, since an individual has the target characteristic.

**Table 4:** Means, standard deviations, and one-way analyses of variance in personality traits, alexithymia, and post-traumatic stress (*N* = 506).

	No Symptoms	Brain Fog	Breath Impaired	Sensory Disorders	Multiple Disorders	ANOVA One-Way Test
	M (SD)	M (SD)	M (SD)	M (SD)	M (SD)	
**Personality traits**						
PID-5-BF NA	1.33 (0.68)	1.63 (0.67)	1.63 (0.66)	1.59 (0.70)	1.55 (0.56)	F(4, 501) = 3.99, *p* = 0.004, η^2^ = 0.034
PID-5-BF DE	0.83 (0.59)	1.10 (0.65)	1.02 (0.67)	1.05 (0.65)	0.85 (0.67)	F(4, 501) = 3.64, *p* = 0.006, η^2^ = 0.028
PID-5-BF AN	0.56 (0.47)	0.55 (0.49)	0.53 (0.49)	0.59 (0.51)	0.50 (0.57)	F(4, 501) = 0.33, *p* = 0.857, η^2^ = 0.003
PID-5-BF DI	0.74 (0.62)	0.83 (0.64)	0.77 (0.55)	0.87 (0.67)	0.77 (0.62)	F(4, 501) = 0.74, *p* = 0.564, η^2^ = 0.006
PID-5-BF PS	0.63 (0.58)	0.82 (0.59)	0.89 (0.67)	0.92 (0.66)	0.78 (0.61)	F(4, 501) = 3.40, *p* = 0.009, η^2^ = 0.026
**Alexithymia**						
TAS-20 DIF	2.31 (1.00)	3.03 (1.03)	3.34 (1.01)	2.98 (1.08)	3.07 (0.85)	F(4, 501) = 14.14, *p* < 0.001, η^2^ = 0.101
TAS-20 DDF	2.47 (0.97)	2.64 (0.94)	2.77 (0.85)	2.67 (0.93)	2.52 (0.98)	F(4, 501) = 1.56, *p* = 0.184, η^2^ = 0.012
TAS-20 EOT	2.61 (0.94)	2.72 (0.96)	2.85 (1.04)	2.69 (0.95)	2.34 (0.74)	F(4, 501) = 2.68, *p* = 0.031, η^2^ = 0.021
**Post-Traumatic Stress**						
IES-R Avoidance	1.37 (0.89)	1.99 (0.92)	2.17 (0.98)	1.94 (1.01)	1.75 (0.80)	F(4, 501) = 10.66, *p* < 0.001, η^2^ = 0.078
IES-R Intrusion	1.62 (0.95)	2.46 (0.90)	2.68 (1.00)	2.32 (1.08)	2.23 (0.95)	F(4, 501) = 17.29, *p* < 0.001, η^2^ = 0.121
IES-R Hyperarousal	1.72 (1.05)	2.68 (0.90)	2.85 (0.92)	2.54 (1.01)	2.42 (0.88)	F(4, 501) = 21.95, *p* < 0.001, η^2^ = 0.149

Notes: PID-5-BF = Personality Inventory for DSM-5 Brief Form, NA = Negative Affectivity, DE = Detachment, AN = Antagonism, DI = Disinhibition, PS = Psychoticism, TAS-20 = Toronto Alexithymia Scale-20, DIF = Difficulty in Identifying Feelings, DDF = Difficulty in Describing Feelings, EOT = Externally Oriented Thinking, IES-R = Impact of Event Scale Revised.

**Table 5:** Odds ratio from the multinomial logistic regression model for the post-COVID-19 symptom classes (*N* = 506).

	Brain Fog OR [95%CI]	Breath Impairment OR [95%CI]	Sensory Disorders OR [95%CI]	Multiple Disorders OR [95%CI]
(Intercept)	2.25	1.11	1.35	0.68
PID-5-BF NA	0.93 [0.64; 1.36]	0.79 [0.52; 1.2186]	0.85 [0.57; 1.29]	0.98 [0.60; 1.55]
PID-5-BF DE	1.43 [0.94; 2.09]	1.00 [0.63; 1.5602]	1.15 [0.72; 1.74]	0.84 [0.47; 1.32]
PID-5-BF AN	0.64 * [0.46; 0.92]	0.65 * [0.44; 0.9849]	0.66 * [0.45; 0.96]	0.68 [0.42; 1.04]
PID-5-BF DI	1.07 [0.77; 1.46]	0.86 [0.59; 1.2470]	1.06 [0.74; 1.49]	0.98 [0.64; 1.47]
PID-5-BF PS	0.94 [0.60; 1.46]	1.11 [0.68; 1.8353]	1.40 [0.88; 2.27]	1.16 [0.65; 1.98]
TAS-20 DIF	2.05 ** [1.23; 2.99]	3.17 ** [1.79; 5.1122]	1.90 ** [1.07; 2.84]	3.87 ** [1.91; 6.17]
TAS-20 DDF	0.69 [0.47; 1.02]	0.72 [0.46; 1.14]	0.76 [0.50; 1.17]	0.75 * [0.45; 1.25]
TAS-20 EOT	0.74 [0.52; 1.05]	0.79 [0.53; 1.17]	0.70 [0.48; 1.02]	0.44 * [0.28; 0.71]
IES-R Avoidance	0.88 [0.51; 1.35]	0.88 [0.49; 1.46]	1.04 [0.58; 1.64]	0.85 [0.41; 1.37]
IES-R Intrusion	1.05 [0.62; 1.97]	1.32 [0.70; 2.70]	0.88 [0.49; 1.76]	0.98 [0.51; 2.22]
IES-R Hyperarousal	2.54 ** [1.45; 4.39]	2.33 * [1.19; 4.44]	2.16 * [1.17; 3.94]	1.74 [0.86; 3.54]
**Nagelkerke’s Pseudo-R2**	0.24			

Notes. ** *p* < 0.001; * *p* < 0.05. PID-5-BF = Personality Inventory for DSM-5 Brief Form, NA = Negative Affectivity, DE = Detachment, AN = Antagonism, DI = Disinhibition, PS = Psychoticism, TAS-20 = Toronto Alexithymia Scale-20, DIF = Difficulty in Identifying Feelings, DDF = Difficulty in Describing Feelings, EOT = Externally Oriented Thinking, IES-R = Impact of Event Scale Revised.

## Data Availability

The data presented in this study are available on request from the corresponding author. The data are not publicly available due to department policy.
